# Neural Network Ensembles for Sensor-Based Human Activity Recognition Within Smart Environments

**DOI:** 10.3390/s20010216

**Published:** 2019-12-30

**Authors:** Naomi Irvine, Chris Nugent, Shuai Zhang, Hui Wang, Wing W. Y. NG

**Affiliations:** 1 School of Computing, Ulster University, Co. Antrim, Northern Ireland BT37 0QB, UK; cd.nugent@ulster.ac.uk (C.N.); s.zhang@ulster.ac.uk (S.Z.); h.wang@ulster.ac.uk (H.W.); 2 School of Computer Science and Engineering, South China University of Technology, Guangzhou 510640, China; wingng@ieee.org

**Keywords:** human activity recognition, neural networks, ensemble neural networks, model conflict resolution, smart environments

## Abstract

In this paper, we focus on data-driven approaches to human activity recognition (HAR). Data-driven approaches rely on good quality data during training, however, a shortage of high quality, large-scale, and accurately annotated HAR datasets exists for recognizing activities of daily living (ADLs) within smart environments. The contributions of this paper involve improving the quality of an openly available HAR dataset for the purpose of data-driven HAR and proposing a new ensemble of neural networks as a data-driven HAR classifier. Specifically, we propose a homogeneous ensemble neural network approach for the purpose of recognizing activities of daily living within a smart home setting. Four base models were generated and integrated using a support function fusion method which involved computing an output decision score for each base classifier. The contribution of this work also involved exploring several approaches to resolving conflicts between the base models. Experimental results demonstrated that distributing data at a class level greatly reduces the number of conflicts that occur between the base models, leading to an increased performance prior to the application of conflict resolution techniques. Overall, the best HAR performance of 80.39% was achieved through distributing data at a class level in conjunction with a conflict resolution approach, which involved calculating the difference between the highest and second highest predictions per conflicting model and awarding the final decision to the model with the highest differential value.

## 1. Introduction

Human Activity Recognition (HAR) is a challenging and dynamic research field that has been attracting significant interest in recent years [1], as human activities are intricate and highly diverse. Particularly, sensor-based approaches to HAR have become prevalent in pervasive computing, largely due to advancements with sensing technologies and wireless sensor networks. HAR is a fundamental component in an extensive range of application areas, including connected health, pervasive computing, surveillance systems, human computer interaction (HCI), and ambient assisted living (AAL) in smart home settings. Other notable interest domains include human/object detection and recognition based on object analysis and processing, for example, tracking and detection [2,3], computer engineering [4], physical sciences [5], health-related issues [6], natural sciences, and industrial academic areas [7]. Notably, the progression of AAL technologies is becoming vital, due to the continuously increasing cost of healthcare provision, the aging population, and the need to support “aging in place”. In this domain, several dedicated smart home projects have been aimed at AAL for the elderly and disabled, for example CASAS [8], Gator Tech [9], MavHome [10], DOMUS [11], and Aware Home [12]. These environments all employ a large number of sensors that capture activity data via a range of sensor modalities. They possess the common aim of supporting smart home inhabitants in carrying out activities of daily living (ADLs) and providing them with non-intrusive, AAL environments to promote their independence and quality of life. ADL monitoring in smart environments is an important aspect to consider for assessing the health status of inhabitants, therefore the automatic detection of these activities is a significant motivation for conducting HAR research [13]. Various sensors are available for the purpose of image object capturing and processing, including binary sensors, digital cameras, and depth data in image analysis fields [14,15].

Sensor-based approaches to HAR can be deemed generally within two categories: data-driven or knowledge-driven. Data-driven approaches make use of datasets to learn activity models through applying machine learning and data mining techniques [16], whereas knowledge-driven approaches build activity models through exploiting rich prior knowledge in the domain of interest [17]. This work focuses on data-driven approaches to HAR and addresses the current challenges of their application to openly available datasets. Within the context of this work, the availability of openly available datasets prompted focus on data-driven approaches, whilst an awareness of the difficulties in accessing domain knowledge averted attention away from knowledge-driven approaches. Nevertheless, data quality is a substantial consideration as data-driven approaches depend on good training data, however, in the realms of HAR, a shortage of high quality, large-scale, and accurately annotated HAR datasets exists for recognizing ADLs within smart environments [18]. This work avails of low-quality data and emphasizes that good practice concerning data preparation can help improve HAR performance. In relation to this, it has been observed that many machine learning algorithms rely on large amounts of data during the training phase to achieve the desired generalization capabilities [18].

Ensemble learners have been explored widely, due to their ability to improve machine learning performance [19], with the main motivation being the desire to improve generalization capabilities [20]. By combining a set of imperfect models, the acknowledged limitations of individual learners can be more efficiently managed, in that the errors recognized in each component can be minimized as an ensemble, through the implementation of effective combination approaches [20].

In this paper, contributions include improving the quality of an openly available HAR dataset for the purpose of data-driven HAR, since it has been observed that data quality is a substantial consideration for data-driven approaches to HAR, as well as proposing a new ensemble of neural networks as a data-driven HAR classifier. Furthermore, various approaches to resolving conflicts that occur between base models in ensemble classifiers are investigated, and the effects of various data distributions that form the complement class per model are analyzed, as each model in the ensemble contains unique classes. It has been observed that the various data distributions to generate the complement class per model greatly impact the number of conflicts arising between the base models, thus demonstrating that the effective generation of these classes is an important consideration. The importance of adhering to good data preparation practices is also highlighted, as restructuring and balancing the data has supported and notably improved HAR performance. 

The remainder of the paper is structured as follows. Section 2 provides an overview of HAR and describes ensemble approaches to activity classification. Following this, Section 3 describes the dataset used in this study and issues identified with the data. Section 4 provides the methods and materials implemented. Results are then presented and discussed in Section 5, followed by conclusions and future work in Section 6. 

## 2. Related Works

This Section presents relevant background information and related works. Section 2.1 provides information relating to HAR within smart home settings, Section 2.2 describes neural networks with regards to their recent use for HAR tasks and Section 2.3 describes ensemble learners with particular consideration to ensemble generation and integration techniques.

### 2.1. Human Activity Recognition (HAR)

HAR is concerned with the ability to recognize and interpret human activities automatically through the deployment of sensors and the processing of the data they generate [21]. Various approaches to recognizing activities within smart environments have been explored, including the extensive use of wearable devices [22,23] and video-based approaches [24], which is largely due to the increased accessibility of these technologies. Nevertheless, these approaches have associated limitations to consider, including concerns with ethics, comfort, privacy invasion, and obtrusiveness. For example, it has been reported that many elderly inhabitants in AAL scenarios are often reluctant and unwilling to continuously adopt the use of body-worn sensors, in addition to expressing reluctance to the installation of video-based monitoring [25]. Consequently, in an attempt to address the identified concerns and prevent user acceptance issues, binary sensors deployed in the surrounding environment are becoming increasingly promising for long-term activity monitoring in the ubiquitous computing domain, as these devices eliminate the privacy concerns identified with other approaches to HAR, whilst also being non-invasive to smart home inhabitants [16].

Binary sensors have been used in a recent HAR study conducted by [26] to recognize nine ADLs, such as cleaning, cooking and sleeping, performed by four smart home inhabitants. The sensors deployed included motion detectors integrated within, or attached to, smart appliances. These also incorporated ON/OFF states for cleaning appliances, e.g., a vacuum, ceiling lights, cooking heaters, TV and PC, as well as OPEN/CLOSE states for kitchen appliances such as the fridge. The chosen classifier was a Random Forest model which achieved 68% accuracy, however, the researchers suggested this figure could be increased by applying more effective methods. In addition to this, in [27], binary sensors were deployed within a home monitoring environment to recognize four basic activity classes, namely relaxing, preparing a meal, eating, and transitioning from bed to toilet. A Deep Convolutional Neural Network (DCNN) was proposed for activity classification, where the binary sensor data generated by four door sensors and 31 passive infrared (PIR) motion sensors were converted into representative activity images. The images generated were used to train the DCNN model which obtained an accuracy of 99.36% in recognizing the four ADLs observed in the study. Although this approach performed significantly well, a greater number of activity classes could have been explored. Another study conducted by [28] explored the potential of ADL recognition using neural networks within a smart home setting. Experiments involved the design and implementation of recurrent (RNN) and convolutional (CNN) neural networks to recognize activities, e.g., cooking, bathing and sleeping. Data acquired through the deployment of binary sensors consisting of pressure sensors, reed switches, float sensors and PIR motion sensors, was used to train the various neural network classifiers, with results showing that the RNN and CNN models significantly outperformed other common classifiers during comparisons achieving 89.8% and 88.2% accuracies, respectively.

HAR requires a feature extraction stage where a set of features are chosen as inputs to a classification model in order to represent the activities being detected. Various state-of-the-art features have been determined for HAR, however, these vary depending on the sensors used to capture activity data. For example, in the realms of wearable technologies that produce accelerometry data, extracting the maximum, minimum, and range features are beneficial in differentiating between activities that comprise movements of varying ranges [29]. Additionally, calculating the signal magnitude area (SMA) of an accelerometry signal has proven advantageous in differentiating between static and dynamic activities [30]. Alternatively, considering the vision-based HAR domain, visual objects can be represented, for example, using local descriptors [31] or calculating the centroids from the contour of depth silhouettes [32]. In this domain, features are commonly extracted with a template-based approach, for example, through human silhouette representations, or a model-based approach, i.e., where the body is defined by a skeleton-based outline with joint points used as feature representation [33].

### 2.2. Neural Networks 

Neural Networks (NNs) are discriminative models that have been attracting attention recently and are becoming a popular classifier for activity recognition tasks [34]. The Multilayer Perceptron (MLP) is a notable type of feed-forward NN often used for activity recognition tasks [35,36,37,38]. They are capable of modelling complex, non-linear relationships and provide an alternative approach to pattern recognition, which is valuable for application in the HAR domain [35]. NNs require high computational capacities which had restricted their use previously, however, due to recent advancements in technology, more complex architectures are being explored with potential to offer better performance and support [39]. In [37], various approaches to recognizing 11 common ADLs were explored, including the use of a single hidden layer NN, a deep NN architecture, and a fuzzy rule-based approach. The shallow NN performed best with an accuracy of 97.72%, followed by the deep NN approach with 96.59%, with the researchers stating the potential of deep NNs had not been shown during the study, whilst also stating that this could be due to insufficient amounts of training data. IN addition to this, in a study conducted by [40], an efficiency investigation was carried out which compared HAR performance using shallow to deep NN approaches. The shallow NN outperformed the convolutional neural network (CNN) on the evaluated HAR datasets, with the shallow NN achieving 99.2% on the WARD data and 96.7% on the UCI_DB data [41], in comparison to 97.7% and 94.2% with the CNN model, respectively. Conclusions of this study stated that the optimal choice for HAR tasks is the use of shallow NNs with two or three layers, rather than the implementation of more complex architectures, particularly if the dataset contains a small number of training samples.

### 2.3. Ensemble Learners 

A technique often used to improve classification performance is to combine multiple models together, i.e., to create an ensemble method, rather than relying on the performance of a single model [29]. Ensemble learning involves two key considerations: ensemble generation and ensemble integration [42]. The generation phase includes generating the base models and determining the size of the ensemble. If the models created are achieved using a consistent induction algorithm, it is known as a homogeneous approach, whereas a heterogeneous approach involves creating models using various different algorithms [43]. In [44], a heterogeneous ensemble approach was implemented to recognize various activities within the CASAS smart home testbeds. The ensemble included four base classifiers, which included a Hidden Markov Model (HMM), a NN, a Support Vector Machine (SVM), and Conditional Random Fields (CRF). The results were promising and revealed performance improvements over the use of a single classification model. Further to this, [45] implemented an ensemble classification approach to activity recognition using several heterogeneous base classifiers. The five common base classifiers included an SVM, Decision Tree (DT), kNN, NN, and Naïve Bayes. Results demonstrated that the ensemble approach, combined through majority voting, performed extremely well in classifying twelve activities. As for homogeneous approaches, [46] proposed an ensemble of random forest learners with the aim of generating a more accurate, stable classifier to recognize activities from the PAMAP physical activity dataset. Activity recognition performance was very high, and the generalization capability of the produced classifier had improved significantly. In [47], multiple HMM base models were combined using a decision templates method to recognize activities collected by a smartphone-embedded triaxial accelerometer. Their approach addressed the interclass similarity and intraclass variability HAR challenges, with results showing the ensemble generated performed significantly well with data representing six activity classes and collected by 30 participants. In addition to this, [48,49,50,51] proposed homogeneous ensemble approaches for HAR. An observation has been made that less research effort exists on heterogeneous ensembles due to more difficulties arising in controlling interactions between the various learning processes [43]. More recently, researchers have been exploring ensemble learners on the basis of deep learning approaches. For example, [52,53,54] proposed ensemble deep learning techniques for HAR, which revealed positive results and robustness. Nevertheless, NNs, and more specifically, deep learning techniques, require a large number of training samples to enhance their performance [55]. 

#### 2.3.1. Ensemble Generation 

During ensemble generation, data partitioning is a commonly considered approach aimed towards diversifying the input data of the base models, so that the subspaces of inputs become complementary [56]. Boosting and Bagging are two common data partitioning ensemble methods used to combine multiple classification models that have been trained on different subsets of the training data [29]. Boosting involves the combination of multiple base classifiers to generate a strong committee classifier that may provide significantly enhanced performance in comparison to the base classifiers, achieved through reweighting the misclassified data samples and therefore boosting their performance [29]. SMOTEBoost and RUSBoost are adaptations of the known AdaBoost approach, where random undersampling or SMOTE is applied to the base classifiers training data, along with the reweighting phase in accordance with the AdaBoost algorithm, as demonstrated in a study conducted by [57]. Both SMOTEBoost and RUSBoost inject a great degree of arbitrariness through generating or removing instances, resulting in improved robustness to noise [57]. Bagging, on the other hand, averages the outputs produced by each base model, where each model is trained on different training sets consisting of data generated through sampling with replacement [42]. Examples of well-known bagging-based approaches include OverBagging, UnderBagging, and SMOTEBagging. Particularly, SMOTEBagging has been recommended for handling multi-class imbalanced data problems where the instances within each bag are significantly diverse [58]. In a recent study [59], two bagging-based hybrid methods were proposed to deal with imbalanced datasets, namely, ADASYNBagging and RSYNBagging. The ADASYNBagging approach uses the bagging algorithm in conjunction with the ADASYN-based oversampling method, whereas the RSYNBagging approach uses the ADASYN-based oversampling method as well as random undersampling alongside the bagging algorithm. The performances of the proposed hybrid approaches were compared against UnderBagging and SMOTEBagging techniques and evaluated on twelve datasets, with promising experimental results obtained. The benefits of the proposed hybrid approaches were demonstrated, as they outperformed the benchmark methods on eight of the twelve datasets evaluated. Another approach considered during ensemble generation is to manipulate the inputs of the base classifiers at a feature level, for example, training the base models on various different subsets of features [56].

#### 2.3.2. Ensemble Integration 

The ensemble integration phase determines how the predictions produced by the base models should be integrated together to increase performance by obtaining a single outcome [42]. Multiple fusion strategies exist and can be considered at a class label level, a trainable level, or a support level, according to [55]. The class label fusion technique involves each of the base classifiers voting for a certain class, then the final output is decided upon through either a majority voting or weighted majority voting strategy. Majority voting decides on the final output prediction based on the class that has been chosen most often or unanimously by the base classifiers, whereas weighted majority voting assigns weights to each model, often based on their performances, where the classifier with the highest output after weight assignments wins the overall prediction [55]. In a study conducted by [60], majority voting was implemented to decide upon the final outputs of an ensemble approach based on AdaBoost. Three weak learners were used, namely a Decision Tree, Logistic Regression, and Linear Discriminant Analysis (LDA). In addition to AdaBoost, Bagging and Stacking methods were also explored, with the best performance produced by the Bagging approach. Another study [46] used weighted majority voting with an ensemble of Random Forest classifiers. Each classifier was assigned different weights per activity, with the final outcome attained through combining the classification outcomes from each base model via the weighted votes. 

Fusion techniques at a trainable level consider the chosen fusion weights during the learning process and implement optimization strategies to increase classification performance whilst also reducing computation cost [55]. These include weighted summations of hypotheses, where higher weights are assigned to those with lower error rates and the Dempster–Shafer theory to handle uncertainty in the decision-making process. In [61] the outputs of various SVM classifiers, trained on different input feature subsets, were subsequently combined using the Dempster-Shafer fusion rule. The four-step process included creating decision templates for all training instances, calculating the proximity between decision templates and classifier outputs, computing the belief degrees for each output class, and finally, applying the Dempster rule to combine the degrees of belief derived from each base classifier. 

Finally, support function fusion involves computing an output decision score for each base classifier, which is derived from the estimated likelihood of a class [56]. This estimation can be computed as an a posteriori probability attained through probabilistic models, using fuzzy membership functions, or through combining NN outputs according to their performance. In [62], five classifiers were combined using an average of probabilities fusion method to recognize six activities. This method used the average of the probability distributions for each base classifier to make a final decision, achieving the best HAR performance in comparison to a majority voting approach that was also implemented during this study. Another study [63], implemented a support function fusion where a Naïve Bayesian fusion method was compared to a majority voting approach to fuse several HMM base models. The Naïve Bayesian approach involves calculating the posterior probability of the HMM outputs, which achieved the best activity recognition performance during the study.

After reviewing the literature, we focus on ensemble learning for HAR in this work, due to their perceived benefits, rather than relying on the performance of a single model. Particularly, an ensemble of NNs are explored, although, due to a lack of high quality data in ADL datasets and a low quantity of data, it was decided to employ lighter weight models rather than exploring deeper architectures. The literature has shown that shallow NNs have previously achieved similar performance to deep NN architectures for HAR tasks, with provided recommendations to use shallow architectures particularly in cases where a small number of training samples are available. As outlined, there has been less effort made with heterogeneous ensembles in the research community due to difficulties existing in controlling interactions between the various learning processes, consequently, this work focuses on a homogeneous approach to generating an ensemble of NNs. As stated in [64], one of the crucial problems to consider with ensemble learning is the combination rule employed to determine a final class decision amongst the base models. In this work, a support function fusion method is used to integrate base models, and various approaches to effectively resolve conflicts that occur between the base models are investigated to determine a final output decision.

In summary, this section contained detailed relevant background information and related works. The recent potential of NNs for HAR and pattern recognition problems was presented, which demonstrated that shallow architectures are preferred in scenarios where the dataset contains a small number of training samples. Ensemble approaches to activity recognition were also discussed because of their recent performance in the HAR domain. As mentioned, this work focuses on data-driven approaches to HAR, thus the importance of data quality in relation to those is considered in Section 3, along with a description of the dataset used to conduct experiments. 

## 3. Dataset for Data-Driven HAR

An overview of the HAR data is presented in this section with an emphasis on the quality of data acquired. The UCAmI Cup challenge is also described, as the dataset used in this study was derived from this competition. Section 3.1 outlines details of the original dataset, Section 3.1.1 highlights the problems identified, and Section 3.2 details the restructured dataset created as a result of the encountered problems and to demonstrate more realistic capabilities of binary datasets for HAR in smart environments.

Data collection is becoming a critical concern among the countless challenges in machine learning, largely due to limited amounts of training data being available to researchers in their respective fields and the quality of the data being collected [65]. In the realms of machine learning, it is known that the majority of effort and time is consumed through preparing the data, which involves data collection, cleansing, interpretation, and feature engineering [65]. Data quality is an imperative consideration in applications of data-driven approaches to HAR, as the performance of models are largely dependent on the quality of training data. Noise can be introduced during data collection by the participants and/or sensors which adversely affects the performance of data-driven techniques [66]. Common issues include missing or erroneous values and mislabeled data [67]. Data cleansing is known as the process of removing inconsistencies or errors, such as outliers and/or noise from a collection of data [68]. According to [66], addressing the presence of outliers and noise is vital as their existence can substantially influence experimental results produced by data-driven approaches. Nevertheless, an unclear border is often present between normal and abnormal data, where a considerably large “gray area” may exist [69]. In supervised learning, noise can transpire at an attribute or class level. In [70], an effort was made to evaluate the impact of noise on classification performance on 17 datasets, generated within various domains. Each dataset was manually introduced to various levels of noise to investigate how it affected model performance. Their findings demonstrated that as noise levels increase, performance decreases, and that attribute noise is generally less harmful than class noise. Furthermore, [71] compared and evaluated how well several classifiers performed with noisy, poor quality data. Conclusions stated that robustness to noisy data and classification performance varied significantly amongst the algorithms observed, with the Random Forest and kNN models proving most resilient to noise. 

The data used in this study was generated for the 1st UCAmI Cup challenge, where participants were invited to use their tools and techniques to analyze a HAR dataset with the aim of achieving the highest accuracy on the unseen test set. In [72], the challenge organizers describe the UCAmI Cup dataset comprehensively. Knowledge-driven rule-based approaches outperformed the data-driven approaches to the activity recognition problem, with many of the participants reporting issues and limitations found within the data [73,74,75,76]. The approach implemented by [73] involved a domain knowledge-based solution inspired by a Finite State Machine, achieving 81.3% accuracy. In [74], a hybrid model was proposed using a hidden Markov chain and logic model. The researchers combined their knowledge-driven and probabilistic models using a weighted averaging method, however, they reported that they had expected a better result than 45.0% accuracy on the test set. In addition to this, [75] used a Naïve Bayes approach with emphasis on location-aware, event-driven activity recognition. The applied method interpreted events as soon as they became available in real-time, omitting the need of an explicit segmentation phase, and generated activity estimations using an activity prediction model. Reported results show mean accuracies of around 68%, with the researchers stating that given the high number of activity classes, the outcome achieved was reasonable. Another approach implemented in [76] used various common machine learning algorithms, including a Decision Tree, Nearest Neighbour, Support Vector Machine, and three ensemble approaches including Random Forest, Boosting, and Bagging. The researchers reported a training set accuracy of 92.1%, however, their approach achieved 60.1% on the provided test data which demonstrated poor generalization. Their suggested cause for the low outcome was the high imbalance of classes in the training set, and they stated that the training algorithm required more labelled training data to perform better.

### 3.1. UCAmI Cup Dataset

**Figure 1:**
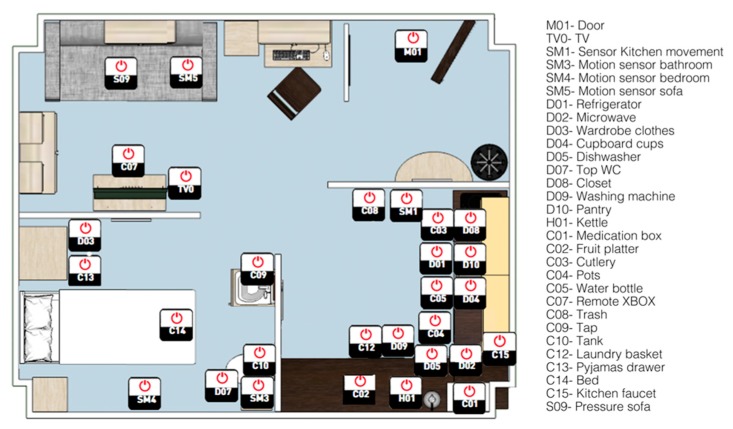
Location of Binary Sensors in the UJAmI Smart Lab [72].

The HAR dataset was collected over 10 days by researchers in the UJAmI Smart Lab [72]. The UJAmI Smart Lab is divided into five regions: an entrance, a workplace, a living room, a bedroom with an integrated bathroom, and a kitchen, which measures approximately 25 square meters combined, as presented in Figure 1. The dataset was captured by a single male inhabitant completing morning, afternoon, and evening routines, representing 246 occurrences of 24 activity classes, as presented in Table 1. The training set consisted of 7 days of labelled data, with the remaining 3 days of data being provided as an unlabeled test set. 

A set of 30 binary sensors consisting of magnetic contact switches, PIR motion detectors, and pressure sensors were deployed in the UJAmI Smart Lab to capture human interactions within the environment, as presented in Figure 1. The two changeable states of the magnetic contact switches were open/close, which were attached to, or integrated within, doors and objects, such as the medication box. The motion detectors generated and recorded movement/no movement states to identify whether an inhabitant had moved in or out of the 7-meter sensing range. Finally, the pressure sensors deployed generated either pressure/no pressure states and was present in the bed and the sofa to detect any interactions. A comprehensive description of each binary sensor is presented in Table 2. 

#### 3.1.1. Data Challenges 

A number of issues were identified with the original binary dataset that hindered the performance of recognizing ADLs in a smart environment setting. These included:

- Number of classes. The number of classes in the original dataset were very high given the low number of instances per activity and low amount of data overall. As discussed previously, data-driven approaches rely on large amounts of good quality data. Furthermore, certain classes were too closely related to one another to recognize with binary data alone. For example, the following activities relied on one door sensor: entering the smart lab, leaving the smart lab, and having a visitor to the smart lab. Binary sensors are limited in inferring activities in that they provide information at an abstract level [77], therefore Act08 eating a snack was difficult to distinguish compared to Act03 prepare breakfast, Act04 prepare lunch, and Act05 prepare dinner, as these activities all used similar sensors. Thus in order to capture activities at a finer level, the presentation and interpretation of binary data often requires further knowledge of the environment [78]. This issue was discussed by a UCAmI Cup participant in [75], where conclusions had stated that their achieved activity recognition performance was reasonable given the large number of activity classes present in the dataset.
- Imbalanced dataset. The distribution of instances per class in the original dataset were highly diverse, which may have caused minority classes to be overlooked by the classification model. For example, Act19 wash dishes was represented by 13 instances of data, whereas other activities such as Act17 brush teeth had more than 100 instances. Furthermore, the distribution of instances per class in the provided training and test sets were highly varied. For example, Act09 was very under-represented in the training set, yet the test set included a large number of Act09 instances. Noteworthy, Act09 also produced very similar sensor characteristics to Act12, which was problematic in the initial experiments, as the training set included large amounts of Act12 data. This issue was discussed in [74], where researchers stated that their approach also found difficulty in classifying Act12, due to the poor representation of this activity in the training set, and suggested that the data should be better distributed to improve HAR performance.
- Quantity of data. As previously stated, data-driven approaches require lots of data during the training phase to learn activity models and to ensure these models can generalize well to new data. NN require lots of data to learn complex activity models [79], though the original dataset was relatively small. Thus, more labelled training data could have improved initial experiments. In [76], UCAmI Cup participants suggested the cause for their low HAR performance was the high imbalance of classes in the training set and stated that the training algorithm required more labelled training data to perform better.
- Missing sensors. Act21 work at table had no binary sensor located near the table to distinguish this activity, as presented in Figure 1. This issue caused confusion as the sensor firing for Act21 in the labelled training set was seen to be a motion sensor located in the bedroom, which is irrelevant to Act21 and therefore seen as erroneous. In addition to missing sensors, there were also missing values from sensors that were expected to fire during certain activities. As previously stated, some researchers participating in the UCAmI Cup challenge reported that they found missing values or mislabeling of some activities within the training set. In [73] this issue was discussed, where participants stated that during one instance of Act10 enter the smart lab, the only binary sensor that is expected to fire (M01), does not change states.
- Interclass similarity. This is a common HAR challenge that occurs when certain activities generate similar sensor characteristics, though they are physically different [80]. Table 3 shows the activities that produced similar sensor characteristics, resulting in difficulties arising in discriminating between these activities during classification.

As a result of the various problems identified with the dataset, it was decided to restructure the data to reveal the potential of using binary sensors alone within smart environments. 

### 3.2. Restructured Dataset

**Figure 2:**
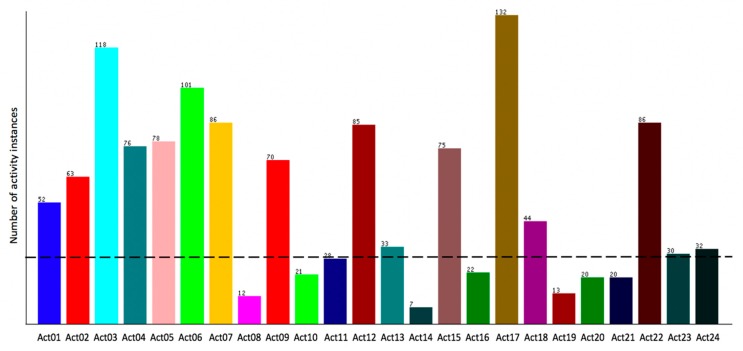
Distribution of the 24 UCAmI Cup activity classes with threshold shown.

First, the provided training and test sets were combined to better represent activity classes within the training data. Figure 2 shows the distribution of the combined 10 days of 24 activity classes for all the available data in the UCAmI Cup. As can be viewed in Figure 2, certain classes were very under-represented, with a third of all activity classes containing less than 30 instances. These classes were removed, as they would be under-represented in the training phase and therefore would not generalize well to unseen data. Consequently, 8.82% of instances were removed, which comprised the following classes: Act08, Act11, Act16, and Act19-Act21. An opportunity to combine certain similar activity classes was also identified so that the data could be used effectively. For example Act10, Act13, and Act14 were combined to produce ActN1 door, as they all make use of a single door sensor, and Act09 and Act12 were combined to produce ActN2 watch TV on sofa, as they mainly consisted of the inhabitant sitting on the sofa. Furthermore, Act02 and Act05, Act03 and Act06, and finally Act04 and Act07 were combined to produce ActN3 breakfast, ActN4 lunch, and ActN5 dinner, respectively, as these sets of activities were similar. Table 4 presents the restructured dataset. 

## 4. Proposed HAR Classification Model

The materials and methods implemented are described within this section. The data pre-processing phase is explained, including data segmentation and feature extraction, which are two fundamental aspects of the activity recognition process [80]. Following this, the ensemble approach and conflict resolution techniques are presented. 

### 4.1. Data Pre-Processing

**Figure 3:**
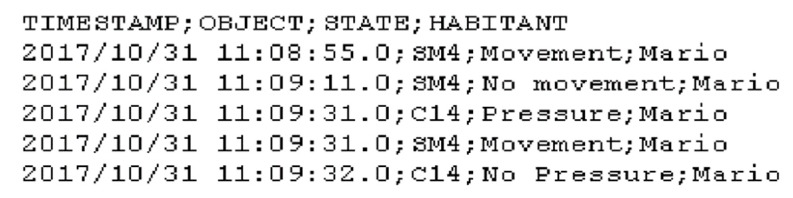
Excerpt from a raw binary data file.

Since the data restructuring process involved combining the provided train and test sets to produce a set of data that better represents activity classes in the training data, it was subsequently required to extract a new test set. Thus, 15% of the data was randomly selected and removed to generate an unseen test set. The raw data files containing data streams produced by binary sensors include a timestamp, the sensor ID, the sensor state, and the inhabitant name, as presented in Figure 3. 

The raw data was segmented into 30-second non-overlapping time windows to identify the segments of data that are likely to contain information regarding activities. Time-based windowing involves dividing the entire dataset equally into time segments that include a fixed quantity of data per window [29]. It is a common approach for segmenting data streams collected through environmental sensors, however, no clear consensus exists for choosing the optimal window size for ADL recognition [81], therefore a 30 second window size was chosen, as this was the regulation adhered to in the UCAmI Cup challenge. A total of 31 features were included, which consisted of one feature per binary sensor and an additional time routine feature representing whether the activity had occurred in the morning, afternoon, or evening, to help distinguish between the similar activities previously outlined. For example, as Act23 go to bed and Act24 wake up use the same pressure sensor located in the bed, the inclusion of a time routine feature can help distinguish these activities due to the human nature of habitually waking up in the morning and going to bed in the evening. 

### 4.2. Ensemble Approach

**Figure 4:**
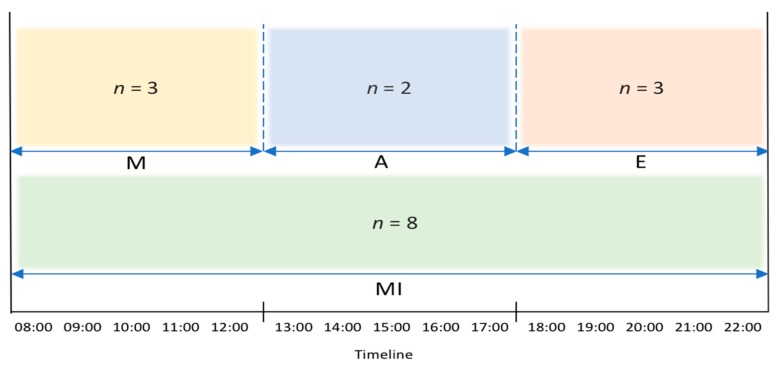
Four base classifiers presented per time routine, where n indicates the number of classes per model. M, A, and E represent the Morning, Afternoon, and Evening models, respectively, and finally MI represents the Mixed model.

Ensemble methods for classification have been explored recently, due to their potential to improve robustness, performance and generalization capabilities in comparison to single model approaches [40]. Our approach consists of four MLPs as base classifiers to generate a homogeneous ensemble method. A model is created per time routine: Morning, Afternoon, and Evening as some activities uniquely occur within specific routines. Additionally, a Mixed model is created to consider activities that occur arbitrarily throughout the day. Figure 4 presents the four base classifiers where n indicates the number of classes per model. M, A, and E represent the Morning, Afternoon, and Evening models, respectively, and finally MI represents the Mixed model. 

Definitions:

Input:

$$X=\;{\left[{\vec{x}}_{1},\;{\vec{x}}_{2},\;\ldots ,\;{\vec{x}}_{M}\right]}^{R}\;\in \;B^{N\times d},$$

where N is the number of instances, d is the number of features, d=31.

$${\vec{x}}_{i}=\left[x_{1}^{1},\;x_{2}^{2},\;\ldots \;,\;x_{i}^{d}\right]\;\mathrm{where}\;x_{i}^{d}\in \left[0,1\right].$$

Output:

$$Y={\left[y_{1},y_{2},\;\ldots ,{\;y}_{N}\right]}^{R}\;\in \left[1,\;\ldots ,\;12\right].$$

Base Models:

Models M_1_, M_2_, M_3_, and M_4_ represent the Morning, Afternoon, Evening, and Mixed base models, respectively, in the proposed ensemble approach.

Given the instance ${\vec{x}}_{i}$ base model output $M_{j}$ is given by

$$f_{i}^{j}=\;f^{j}\left(\varphi^{j}\left(x_{i}\right)\right),$$

where index *j* = [1, … , 4]; $\varphi^{j}\left(x_{i}\right)$ is the input to the activation function of base model $M_{j}$ and $f^{j}$ is the output of each base model $M_{j}$

For simplicity, the output can be represented as $f_{i}^{j}=\left[p_{1}^{j},\;\ldots ,\;p_{m_{j}}^{j}\right],$ where $m_{j}$ represents the number of outputs from base model $M_{j}.$

Predicted class ${\hat{k}}_{i}^{j}\;\in \left[1,\;\ldots ,\;12\right]$ from base model $M_{j}$ is the class represented by the output with maximum *p* values $p_{i}^{j,1}=\max\left[p_{1}^{j},\;\ldots ,\;p_{m_{j}}^{j}\right]$.

The second largest value in the output vector is notated as $p_{i}^{j,2}$. *p* values will be used for later conflict resolution in Algorithms 2–5.

Base Model Compositions:

Universal set C represents the set of all classes of activities; $C^{j}$ represents activity classes represented by the time domain of each base model $M_{j}$

$\tilde{C^{j}}$ is the complement class for base model $M_{j}$ and it combines the activity classes not in the $C^{j}$ denoted below

$$\left\{k\;\in C\;:k\;\notin \;C^{j}\right\}$$

Example: Morning Base Model $M_{1}$ contains activities from classes

$$C^{1}=\left[Act24,\;ActN3\right]$$

$$\tilde{C^{1}}\;=\left[\left\{ActN4,\;Act23,\;ActN5,Act01,Act15,Act17,Act18,Act22,ActN1,ActN2\right\}\right]$$

There are $m_{j}=3$ number of classes, where all but one class, the complement, are in $C^{1}$.

The morning model contains two main activity classes, namely Act24 wake up and ActN3 breakfast, as these activities occur in a typical morning routine. ActN4 lunch, is the only main class within the afternoon model as lunch usually occurs in the afternoon. The evening model contains two main classes, namely Act23 go to bed and ActN5 dinner, as these activities habitually occur in an evening routine. Finally, the mixed model contains seven main activity classes that do not regularly occur within a specific time routine. For example, Act15 put waste in the bin and Act22 dressing are activities commonly performed at any time during the day. The activity class outputs per model are presented in Table 5.

**Figure 5:**
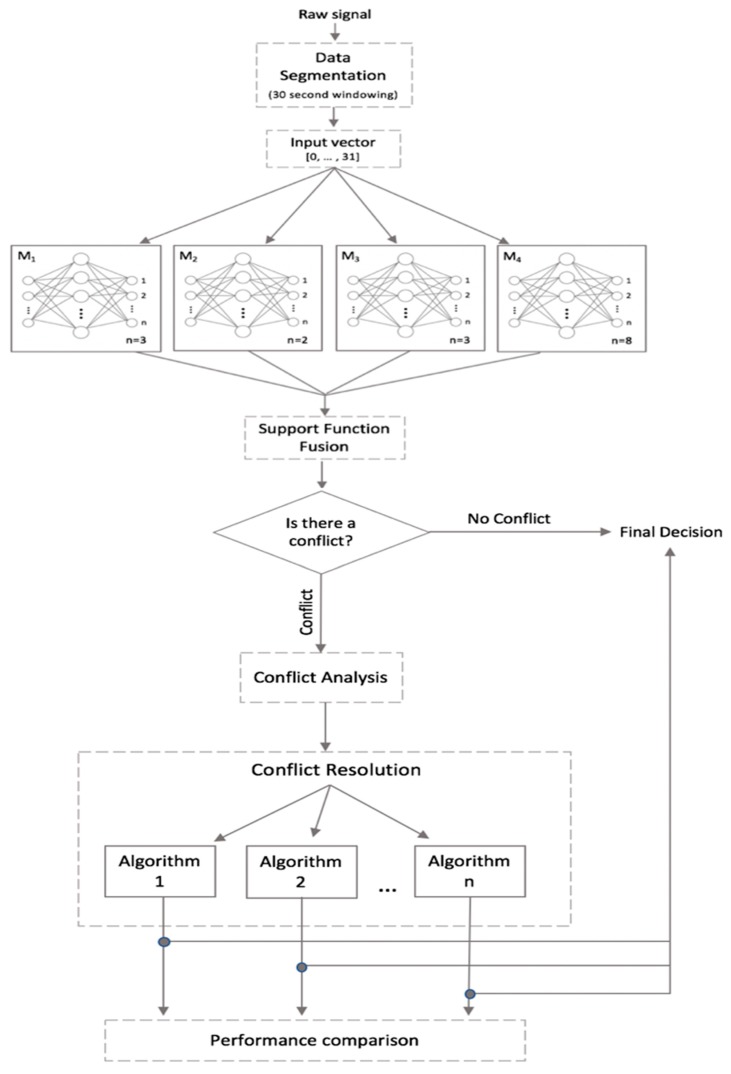
Framework for the homogeneous ensemble approach. *M*_1_, *M*_2_, and *M*_3_ represent the Morning, Afternoon, and Evening models, respectively, and *M*_4_ represents the Mixed model.

A framework for the implemented homogenous ensemble approach is presented in Figure 5, where the conflict resolution approaches are compared. Each base model is presented with an input feature vector consisting of data produced by 30 binary sensors and an additional time routine feature, resulting in a total of 31 input features. Each of the base models produce output predictions derived from the estimated likelihood of each class, which are subsequently combined through the support function fusion [56] during the ensemble integration phase.

Due to each model having no overlapping classes, each needs to be trained with a complement class, which consists of representative activity samples from each of the main classes contained within the remaining models. The aim of this is that each model will be able to identify whether or not new activity instances belong to that model, thus when a model receives an unseen input of an activity class existing within its complement, it should recognize that the activity does not exist as a main class in the model and should, therefore, eliminate itself from the decision process. For example, if the morning model is presented with an activity instance contained in the $\tilde{C^{1}}$ class, e.g., ActN4, as presented in Table 5, it should recognize that ActN4 belongs to the complement class and should therefore exclude itself from the decision making process. To analyze the effects on model conflicts of various data distributions that construct the complement classes per model, we explore two approaches towards generating these classes. Section 4.2.1 explains the generation of the complement class data at a model level, where activity instances are distributed evenly between the remaining models, and Section 4.2.2 explains the generation of the complement class data at a class level, where activity instances are distributed evenly between the remaining classes.

#### 4.2.1. Complement Class Generation at a Model Level 

Distributing instances at a model level involves balancing the complement class data equally between the remaining models. The first step in the process is to calculate how many instances this class should contain, in total. Per model, this is calculated as the average number of main class instances. This total is then divided by the number of remaining models to achieve an equal distribution of instances per model. Following this, the class distributions are calculated by dividing the number of instances per model by the number of main classes within each model. Table 6 presents the distribution of instances at a model level.

#### 4.2.2. Complement Class Generation at a Class Level

Distributing instances at a class level involves balancing the complement class data equally between the remaining classes within the models. As with the previous approach, the first step involves calculating the average number of main class instances per model to attain the total instances for each complement class. Following this, the previously calculated total is divided by the number of remaining classes across the remaining models to achieve an equal distribution of instances per class. Finally, all instances per class were multiplied by 2 to better represent each class. For example, to generate the *M*_1_ complement class, the average number of main class instances was calculated first, resulting in 74. Subsequently, to achieve an equal distribution of instances per class within the complement, 74 was divided by the 10 remaining classes, resulting in 7.4 instances required per class. Finally, to better represent each class during training, this number was multiplied by 2, resulting in 14.8 (15) instances per class. Table 7 presents the distribution of instances at a class level. 

### 4.3. Model Conflict Resolution

As mentioned, support function fusion [56] is explored through combining the output predictions produced by each MLP base model during the ensemble integration phase. The combined predictions are subsequently analyzed to determine whether a single model has chosen the final output, i.e., all models except one had chosen the complement class. If this is not the case, and more than one model has chosen a main class output, a conflict has occurred between these models during the decision making process, as seen in Algorithm 1. We investigate several approaches to the model conflict resolution to determine the final output class per instance.
**Algorithm 1.** Process of finding conflicts between models1:For Each instance ${\vec{x}}_{i}\;\in B^{1\times d}$2: $if\;\exists_{j}\left({\hat{k}}_{i}^{j}\in C^{j}\right)\;\Lambda \exists_{jj}\;\left({\hat{k}}_{i}^{jj}\in \;C^{jj}\;\Lambda \;j\neq jj\right)$3:Then use conflict resolution approaches in Algorithms 2/3/4/5 as there are at least 2 conflicting cases

The first method of resolving conflicts, presented in Algorithm 2, is simply to award the final decision to the model with the highest output prediction. This approach has previously been established as a soft-level combiner [82], as it makes use of the output predictions given by the classifiers as the posterior probabilities of each output class. A limitation of this method, however, is that it provides limited confidence of the output prediction. For example, consider the two largest output values of one base model are 0.56 and 0.54, respectively. If the final class decision is awarded according to the highest output value in this case, there is less confidence in the quality of classification, which implies a less secure output prediction. To overcome this, another technique, presented in Algorithm 3, is proposed to calculate the difference between the highest and second highest predictions per conflicting model, where subsequently the final decision is given to the model with the highest differential value, i.e., this is the model with the strongest class prediction. Following this, the impact of a weighting technique is investigated in Algorithm 4 on the basis of the number of classes per model, as each base model contains a different number of unique classes. This approach considers the output predictions from each conflicting base classifier and the number of classes the base models are trained on, i.e., the output predictions from each base model are multiplied by the number of classes within those base models. For example, if a conflict occurs between model *M*_2_ and model *M*_4_, which contain two and eight classes, respectively, the two class problem may be less complex than the eight class problem, and therefore a lower weighting is specified for *M*_2_. Finally, we explore the potential of another weighted method in Algorithm 5, which builds upon the previous approach. Weightings are implemented on the basis of the number of classes, as well as the training performance per model, i.e., the output predictions from each conflicting base classifier are multiplied by the number of classes in that model and the training performance achieved. According to [83], a base classifier that outperforms other base classifiers in an ensemble approach should be given a higher confidence when deciding upon the final output prediction, as the training performance measure is indicative of the classifiers’ effectiveness in predicting the correct output class. The training performance measure in Algorithm 5 is the classification accuracy obtained by each conflicting model.

Repeated notations:

The largest value in the output vector is notated as $p_{i}^{j,1}\;$.

The second largest value in the output vector is notated as $p_{i}^{j,2}.$
**Algorithm 2.** Conflict resolution approach 1**Input**: ${\vec{x}}_{i}$, base models *M*_r_, *M*_s_**Output**: class $y_{i}$1:$if\;p_{i}^{r,1}\;>\;p_{i}^{s,1}$2:Then $y_{i}={\hat{k}}_{i}^{r}$3:Else $y_{i}={\hat{k}}_{i}^{s}$

**Algorithm 3.** Conflict resolution approach 2 **Input**: ${\vec{x}}_{i}$, base models *M*_r_, *M*_s_**Output**: class $y_{i}$1:
$if\;\left(p_{i}^{r,1}\;-\;p_{i}^{r,2}\right)>\;\left(p_{i}^{s,1}\;-\;p_{i}^{s,2}\right)$
2:Then $y_{i}={\hat{k}}_{i}^{r}$3:Else $y_{i}={\hat{k}}_{i}^{s}$

**Algorithm 4.** Conflict resolution approach 3 **Input**: ${\vec{x}}_{i}$, base models *M*_r_, *M*_s_**Output**: class $y_{i}$1:
$if\;p_{i}^{r,1}\times m_{r}\;>\;p_{i}^{s,1}\times m_{s}$
2:Then $y_{i}={\hat{k}}_{i}^{r}$3:Else $y_{i}={\hat{k}}_{i}^{s}$

**Algorithm 5.** Conflict resolution approach 4 **Input**: ${\vec{x}}_{i}$, base models *M*_r_, *M*_s_**Output**: class $y_{i}$1:$Acc_{train}^{r}$ represents training performance for base model $M_{r}$2:$Acc_{train}^{s}$ represents training performance for base model $M_{s}$3:
$if\;p_{i}^{r,1}\times m_{r}\;\times \;Acc_{train}^{r}\;>\;p_{i}^{s,1}\times m_{s}\;\times \;Acc_{train}^{s}$
4:Then $y_{i}={\hat{k}}_{i}^{r}$5:Else $y_{i}={\hat{k}}_{i}^{s}$

## 5. Results and Discussion

The results show that the class level distribution technique, described in Section 4.2.2, greatly reduces the number of conflicts that occur between the various base models, in comparison to the model level distribution technique, as shown in Table 8. This is due to improved representations of activities within the complement classes per model during the training phase of the base classifiers. For example, with the class level distribution technique activity instances were distributed evenly between classes, therefore evenly representing each activity within the complement class. Contrarily, the model level distribution technique involved balancing the complement class data equally between the remaining models, which meant the class distributions within these models were imbalanced. For example, with the model level distribution technique, the $\tilde{C^{1}}$ complement class contained 24 instances of ActN4 and only 03 instances of Act17, whereas with the class level distribution technique, the $\tilde{C^{1}}$ complement class contained 15 instances each of ActN4 and Act17. Consequently, with the implementation of the latter distribution technique, the base classifiers are stronger at deciding when an unseen instance belongs to their complement class, eliminating themselves from the decision-making process and therefore reducing the number of conflicts that occur. 

**Figure 6:**
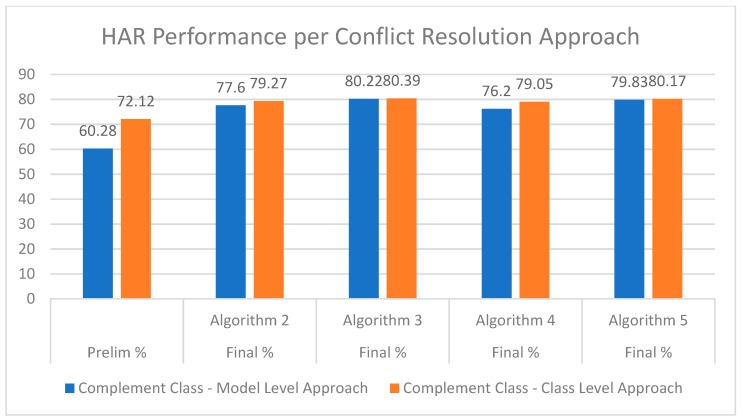
Human Activity Recognition (HAR) performance per conflict resolution approach.

Classification performance from each of the two data distribution techniques were analyzed before and after conflict resolution approaches were applied, as presented in Figure 6. Considering the complement class generation at a model level, the preliminary performance accuracy of 60.28% is much less than that of the complement class generation at a class level, which achieves a preliminary accuracy of 72.12%. This is due to less model conflicts occurring in the latter approach, which shows the base models were stronger during the decision-making process. As for the final accuracies produced after conflict resolution techniques had been applied, the class level approach outperformed the model level approach in all four cases. Finally, overall, the best HAR performance of 80.39% was achieved using complement data generated at a class level in conjunction with the conflict resolution approach presented in Algorithm 3, i.e., resolving conflicts through calculating the difference between the highest and second highest predictions per conflicting model, where the final decision is given to the model with the highest differential value. 

Table 9 presents an analysis of incorrectly classified instances with regards to the first data distribution approach where complement class data was generated at a model level, as discussed previously in Section 4.2.1, whereas Table 10 presents an analysis of incorrectly classified instances with regards to the second data distribution approach, where complement class data was generated at a class level, as discussed previously in Section 4.2.2. The “incorrect” instances reported describe those that were incorrectly classified by the target model, for example, there may not have been any conflicting models, yet the incorrect class was chosen by the base classifier. The number of incorrectly classified instances are important to consider when analyzing the effectiveness of each conflict resolution approach, as these cases would permanently be incorrect, regardless of the application of conflict resolution techniques.

The “right but incorrect” cases are those that were correctly classified by the target base model, although they were not chosen during the final decision-making process after applying the conflict resolution approaches. These cases are considered when evaluating the most effective approach of the four explored, as they could have resulted in a correct classification, given the application of an effective conflict resolution technique. 

The conflict resolution approach presented in Algorithm 3 was the most effective when applied to both data distributions, as there were the lowest number of “right but incorrect” instances (on average 11.3 and 4.7, respectively), closely followed by the approach in Algorithm 5. The lower the number of “right but incorrect” cases helps to determine which conflict resolution approach is most effective in deciding upon which base model should be awarded the final class decision. For example, consider the conflict resolution technique in Algorithm 3 with ensemble approach 2, as presented in Table 10. There were 23.3 conflicts occurring on average (refer to Table 8). Upon analysis of the incorrectly classified instances, 30.4, on average, were incorrectly classified, whereas 4.7, on average, could have been correctly classified, though an incorrect base model won the final decision after applying conflict resolution. Finally, this means that as a result of applying Algorithm 3, an average of 18.6 conflicting cases were correctly resolved, improving the final HAR performance.

**Figure 7:**
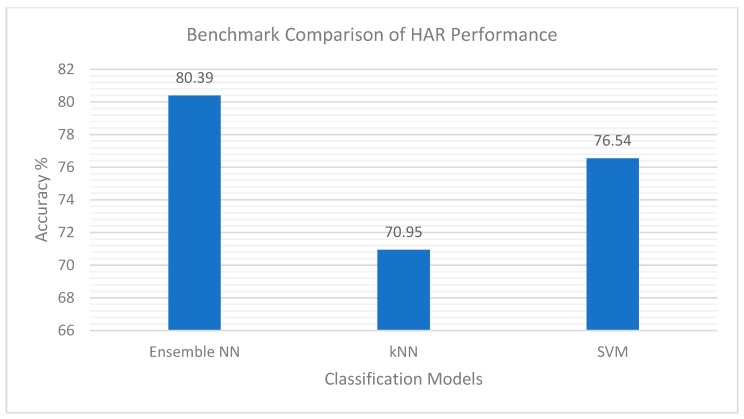
HAR performance of the proposed ensemble Neural Network (NN) approach compared to Nearest Neighbour (kNN) and Support Vector Machine (SVM) classifiers.

As shown in Figure 6, the best HAR performance of 80.39% was achieved using complement data generated at a class level in conjunction with the conflict resolution approach presented in Algorithm 3. Given the non-parametric nature of the neural networks, two non-parametric benchmark classifiers were chosen to evaluate the proposed ensemble approach, namely, Support Vector Machine (SVM) and Nearest Neighbour (kNN) classifiers. The multiclass SVM classifier was an error-correcting output codes (ECOC) model required for multiclass learning, consisting of multiple binary learners. Figure 7 presents the performance of our ensemble approach in comparison to the chosen non-parametric benchmark classifiers. The kNN model achieved an accuracy of 70.95%, whereas the SVM model achieved 76.54%, thus demonstrating that the proposed ensemble approach outperformed both benchmark classifiers.

## 6. Conclusions

In this work, we focused on data-driven approaches to HAR and addressed the current challenges of their application to openly available datasets. We proposed an ensemble approach to recognize ADLs within a smart environment setting, with particular emphasis on exploring various approaches to resolving conflicts that occur between base models in ensemble classifiers and analyzing the effects of various data distributions that generate the complement class per base model. It was observed that distributing data at a class level greatly reduces the number of conflicts that occur between the base models, leading to an increased preliminary performance before the application of conflict resolution techniques. It was also found that the best method of resolving conflicts, in comparison to other approaches explored, is to award the final decision to the model with the highest differential value between the highest and second highest predictions per conflicting model. We evaluated our proposed HAR classification model, the ensemble NN method, by comparing the achieved HAR performance with two non-parametric benchmark classifiers. The ensemble NN method outperformed both benchmark models, demonstrating the effectiveness of the proposed ensemble approach. 

This work is limited in that feature selection techniques were not applied to determine an optimal subset of input features. According to [84], feature selection is an increasingly significant consideration in machine learning, with the primary aim of its application being to reduce the dimensionality in large, multi-dimensional datasets. Thus, future work would involve the application of feature selection techniques to determine the optimal subset of features required for the classification problem. Additionally, this work is limited in that the proposed approach was evaluated on one HAR dataset, therefore future work would involve evaluating the methods on another dataset so that results are not subjective to only the current dataset.

## Figures and Tables

**Table 1:** Activity Classes in the UCAmI Cup Dataset [72], where M, A, and E indicate the Morning, Afternoon, and Evening routines, respectively.

ID	Name	Instances	Routine	ID	Name	Instances	Routine
Act01	Take Medication	52	A, E	Act13	Leave Smart Lab	33	M, A
Act02	Prepare Breakfast	63	M	Act14	Visitor to Smart Lab	7	M, A
Act03	Prepare lunch	118	A	Act15	Put waste in the bin	75	A, E
Act04	Prepare Dinner	76	E	Act16	Wash hands	22	M
Act05	Breakfast	78	M	Act17	Brush teeth	132	M, A, E
Act06	Lunch	101	A	Act18	Use the toilet	44	M, A, E
Act07	Dinner	86	E	Act19	Wash dishes	13	A, E
Act08	Eat a snack	12	A	Act20	Put washing in machine	20	M, A
Act09	Watch TV	70	A, E	Act21	Work at the table	20	M
Act10	Enter Smart Lab	21	A, E	Act22	Dressing	86	M, A, E
Act11	Play a videogame	28	M, E	Act23	Go to bed	30	E
Act12	Relax on the sofa	85	M, A, E	Act24	Wake up	32	M

**Table 2:** Description of binary sensors [72].

ID	Object	Type	States
SM1	Kitchen area	Motion	Movement/No movement
SM3	Bathroom area	Motion	Movement/No movement
SM4	Bedroom area	Motion	Movement/No movement
SM5	Sofa area	Motion	Movement/No movement
M01	Door	Contact	Open/Close
TV0	TV	Contact	Open/Close
D01	Refrigerator	Contact	Open/Close
D02	Microwave	Contact	Open/Close
D03	Wardrobe	Contact	Open/Close
D04	Cups cupboard	Contact	Open/Close
D05	Dishwasher	Contact	Open/Close
D07	WC	Contact	Open/Close
D08	Closet	Contact	Open/Close
D09	Washing machine	Contact	Open/Close
D10	Pantry	Contact	Open/Close
C01	Medication box	Contact	Open/Close
C02	Fruit platter	Contact	Open/Close
C03	Cutlery	Contact	Open/Close
C04	Pots	Contact	Open/Close
C05	Water bottle	Contact	Open/Close
C07	XBOX Remote	Contact	Present/Not present
C08	Trash	Contact	Open/Close
C09	Tap	Contact	Open/Close
C10	Tank	Contact	Open/Close
C12	Laundry basket	Contact	Present/Not present
C13	Pyjamas drawer	Contact	Open/Close
C14	Bed	Pressure	Pressure/No pressure
C15	Kitchen faucet	Contact	Open/Close
H01	Kettle	Contact	Open/Close
S09	Sofa	Pressure	Pressure/No pressure

**Table 3:** Activities producing similar sensor characteristics within the UCAmI Cup data.

Activity Group	Activity Name	Common Sensors
Act10, Act13, Act14	Enter Smart Lab, Leave Smart Lab, and Visitor to Smart Lab	M01 Door
Act23, Act24	Go to Bed and Wake Up	C14 Bed
Act09, Act12	Watch TV and Relax on Sofa	S09 Pressure Sofa SM5 Sofa Motion
Act02, Act03, Act04, Act08	Prepare Breakfast, Prepare Lunch, Prepare Dinner, Prepare Snack	SM1 Kitchen Motion D10 Pantry C03 Cutlery

**Table 4:** Activity classes in the restructured dataset.

ID	Name	Instances	Routine	ID	Name	Instances	Routine
Act01	Take Medication	52	A, E	Act24	Wake up	32	M
Act15	Put waste in the bin	75	A, E	ActN1	Door	61	M, A, E
Act17	Brush teeth	132	M, A, E	ActN2	Watch TV on sofa	155	M, A, E
Act18	Use the toilet	44	M, A, E	ActN3	Breakfast	141	M
Act22	Dressing	86	M, A, E	ActN4	Lunch	219	A
Act23	Go to bed	30	E	ActN5	Dinner	162	E

**Table 5:** Activity class outputs per model.

#output	Model ID	Name	Activity Classes
m_1_ = 3	*M*_1_	Morning	$C^{1}$ = [Act24, ActN3]←2 classes $\tilde{C^{1}}\;$ = [ActN4, Act23, ActN5, Act01, Act15, Act17, Act18, Act22, ActN1, ActN2]←1 class
m_2_ = 2	*M*_2_	Afternoon	$C^{2}$ = [ActN4]←1 class $\tilde{C^{2}}$ = [Act24, ActN3, Act23, ActN5, Act01, Act15, Act17, Act18, Act22, ActN1, ActN2]←1 class
m_3_ = 3	*M*_3_	Evening	$C^{3}$ = [Act23, ActN5]←2 classes $\tilde{C^{3}}$ = [Act24, ActN3, ActN4, Act01, Act15, Act17, Act18, Act22, ActN1, ActN2]←1 class
m_4_ = 8	*M*_4_	Mixed	$C^{4}$ = [Act01, Act15, Act17, Act18, Act22, ActN1 ActN2]←7 classes $\tilde{C^{4}}$ = [Act24, ActN3, ActN4, Act23, ActN5]←1 class

**Table 6:** Model level-distribution of instances for complement class compositions.

Complement	Model Distribution (No. of Instances)	Class Distribution (No. of Instances)	
complement class $\tilde{C^{1}}$ of *M*_1_	Afternoon (24) Evening (24) Mixed (25)	ActN4 (24) Act23 (12) ActN5 (12) Act01 (03) Act15 (03)	Act17 (03) Act18 (04) Act22 (04) ActN1 (04) ActN2 (04)
complement class $\tilde{C^{2}}$ of *M*_2_	Morning (62) Evening (62) Mixed (62)	Act24 (31) ActN3 (31) Act23 (31) ActN5 (31) Act18 (08)	Act15 (09) Act17 (09) Act01 (09) Act22 (09) ActN1 (09) ActN2 (09)
complement class $\tilde{C^{3}}$ of *M*_3_	Morning (27) Afternoon (27) Mixed (27)	Act24 (13) ActN3 (14) ActN4 (27) Act18 (03) Act01 (04)	Act15 (04) Act17 (04) Act22 (04) ActN1 (04) ActN2 (04)
complement class $\tilde{C^{4}}$ of *M*_4_	Morning (24) Afternoon (24) Evening (25)	Act24 (12) ActN3 (12) ActN4 (24)	Act23 (12) ActN5 (12)

**Table 7:** Class level-distribution of instances for complement class compositions.

Complement	Model Distribution (No. of Instances)	Class Distribution (No. of Instances)	
complement class $\tilde{C^{1}}$ of *M*_1_	Afternoon (15) Evening (30) Mixed (105)	ActN4 (15) Act23 (15) ActN5 (15) Act01 (15) Act15 (15)	Act17 (15) Act18 (15) Act22 (15) ActN1 (15) ActN2 (15)
complement class $\tilde{C^{2}}$ of *M*_2_	Morning (68) Evening (68) Mixed (238)	Act24 (34) ActN3 (34) Act23 (34) ActN5 (34) Act18 (34)	Act15 (34) Act17 (34) Act01 (34) Act22 (34) ActN1 (34) ActN2 (34)
complement class $\tilde{C^{3}}$ of *M*_3_	Morning (32) Afternoon (16) Mixed (112)	Act24 (16) ActN3 (16) ActN4 (16) Act18 (16) Act01 (16)	Act15 (16) Act17 (16) Act22 (16) ActN1 (16) ActN2 (16)
complement class $\tilde{C^{4}}$ of *M*_4_	Morning (58) Afternoon (29) Evening (58)	Act24 (29) ActN3 (29) ActN4 (29)	Act23 (29) ActN5 (29)

**Table 8:** Number of conflicts.

	**No. of Conflicts Per Fold**										
	1	2	3	4	5	6	7	8	9	10	Avg.
Complement Class – Model Level Approach	76	57	69	52	49	35	60	45	62	56	56.1
Complement Class – Class Level Approach	21	37	11	13	13	42	29	39	11	17	23.3

**Table 9:** Ensemble approach 1—analysis of incorrect instances.

		**Fold**										
		1	2	3	4	5	6	7	8	9	10	Avg.
Algorithm 2	Incorrect	22	22	21	29	29	20	30	22	20	22	23.7
	Right but Incorrect	17	18	21	12	17	16	9	14	20	20	16.4
Algorithm 3	Incorrect	23	22	21	29	29	22	29	22	20	24	24.1
	Right but Incorrect	10	14	10	9	12	12	9	12	14	11	11.3
Algorithm 4	Incorrect	22	23	21	29	29	22	29	22	20	22	23.9
	Right but Incorrect	31	22	13	23	11	15	23	18	10	21	18.7
Algorithm 5	Incorrect	22	22	21	29	29	22	29	22	20	22	23.8
	Right but Incorrect	14	10	13	7	13	15	9	17	14	11	12.3

**Table 10:** Ensemble approach 2—analysis of incorrect instances.

		**Fold**										
		1	2	3	4	5	6	7	8	9	10	Avg.
Algorithm 2	Incorrect	33	26	35	33	25	32	27	28	40	26	30.5
	Right but Incorrect	6	9	2	6	4	11	8	10	2	8	6.6
Algorithm 3	Incorrect	33	26	35	33	25	31	27	28	40	26	30.4
	Right but Incorrect	5	7	3	2	6	7	6	5	0	6	4.7
Algorithm 4	Incorrect	33	26	35	33	25	31	27	28	40	25	30.3
	Right but Incorrect	8	21	4	3	6	6	11	7	1	5	7.2
Algorithm 5	Incorrect	33	26	34	33	25	31	27	28	40	25	30.2
	Right but Incorrect	8	8	5	2	6	5	6	7	1	5	5.3
